# Receptor-Meditated Endocytosis by Hyaluronic Acid@Superparamagnetic Nanovetor for Targeting of CD44-Overexpressing Tumor Cells

**DOI:** 10.3390/nano6080149

**Published:** 2016-08-18

**Authors:** Kwang Sik Yu, Meng Meng Lin, Hyun-Ju Lee, Ki-Sik Tae, Bo-Sun Kang, Je Hun Lee, Nam Seob Lee, Young Gil Jeong, Seung-Yun Han, Do Kyung Kim

**Affiliations:** 1 Department of Anatomy, Konyang University, Daejeon 302-718, Korea; withreno@konyang.ac.kr (K.S.Y.); leejehun@konyang.ac.kr (J.H.L.); nslee@konyang.ac.kr (N.S.L.); ygjeong@konyang.ac.kr (Y.G.J.); 2 Department of Chemical Engineering, Tsinghua University, Beijing 100084, China; mengmenglin@mail.tsinghua.edu.cn; 3 Physical Therapy, Konyang University, Daejeon 302-718, Korea; leehj@konyang.ac.kr; 4 Biomedical Engineering, Konyang University, Daejeon 302-718, Korea; tae@konyang.ac.kr; 5 Radiological Science, Konyang University, Daejeon 302-718, Korea; bskang@konyang.ac.kr

**Keywords:** superparamagnetic, CD44, hyaluronan, glutamic acid, receptor-meditated endocytosis (RME)

## Abstract

The present report proposes a more rational hyaluronic acid (HA) conjugation protocol that can be used to modify the surface of the superparamagnetic iron oxide nanoparticles (SPIONs) by covalently binding the targeting molecules (HA) with glutamic acid as a molecular linker on peripheral surface of SPIONs. The synthesis of HA-Glutamic Acid (GA)@SPIONs was included oxidization of nanoparticle’s surface with H_2_O_2_ followed by activation of hydroxyl group and reacting glutamic acid as an intermediate molecule demonstrating transfection of lung cancer cells. Fourier transform infrared (FTIR) and zeta-potential studies confirmed the chemical bonding between amino acid linker and polysaccharides. 3-(4,5-dimethylthiazol-2-yl)-2,5-diphenyltetrazolium bromide (MTT) cytotoxicity assay showed that HA-SPIONs-treated cells remained 82.9% ± 2.7% alive at high particle dosage (200 µg/mL iron concentration), whereas GA-SPIONs and bare SPIONs (B-SPIONs) treated cells had only 59.3% ± 13.4% and 26.5% ± 3.1% survival rate at the same conditions, respectively. Confocal microscopy analysis showed increased cellular internalization of HA-SPIONs compared to non-interacting agarose coated SPIONs (AgA-SPIONs).

## 1. Introduction

Superparamagnetic iron oxide nanoparticles (SPIONs) have been widely used as one of the excellent candidates in Nanomedicine including magnetic resonance (MR) imaging contrast agents, carriers for target drug delivery, hyperthermia treatment, etc. Especially, SPIONs can be taken as potential carrier to translocate therapeutic molecules into cellular interiors by active/passive or combined targeting strategies. It is well understood that receptor-mediated endocytosis (RME) and diffusive penetration are two main passive transport pathways [1] by which uptake and intracellular localization of extracellular moieties like nanoparticles and bio-macromolecules [2,3]. Compared to the direct uptake, the size range of RME is much broader, and therefore RME may become a more general pathway.

Mostly, the concept of peripherally conjugation of the specific bio-active molecules to the core SPIONs have been introduced to enhance the delivery efficacy of targeting agents, therapeutic moieties or diagnostic modality. Bare SPIONs have serious limitations such as instability against oxidation in air, dissolution in acids, and agglomeration [4]. Therefore, using protective and “antibiofouling” layers or conjugation of various functional ligands such as peptides, aptamers, antibodies or small molecules that possess strong affinity toward unique molecular signature overexpressed in pathogenic sites on the surface of SPIONs become an important issue to make them more compatible to nanomedicinal applications [5]. The advantage of the active targeting is the selective delivery of target moieties into the specific tumors/pathogenic tissues, in which have differed from the healthy tissues in certain physiological properties, such as temperature, hypoxia, pH, etc.

SPIONs have hydroxyl groups in their peripheral surface, thus it limits the additional conjugation of such bio-active molecules [6]. In addition, one of the major problems encountered in using the nanoparticles in vein is thrombi or blood clot formation (i.e., poor blood compatibility) on their surface [7]. Blood-particle interactions affect the blood-particle compatibility, which involves a complicated blood clotting process (including protein adsorption, platelet adhesion, and activation) and blood coagulation cascades (intrinsic and extrinsic pathways) [8]. Low fibrinogen adsorption and low platelet adhesion are essential for achieving favorable blood compatibility. Hence, surfaces that are “antibiofouling” [9] to fibrinogen adsorption should be modified to prevent platelet adhesion and activation, by adjusting the surface wettability and surface roughness [10,11].

Biologically inert materials are commonly used substances to improve water-dispersity, bio-compatibility and further conjugation of biological modalities, usually proteins or peptides [12]. However, non-specific adsorption of proteins on the surface of nanoparticles occurs quite often once injected into the body, causing undesired side effects such as quick clearance through the reticuloendothelial system (RES) [9]. RES also called macrophage system or mononuclear phagocyte system, is a class of cells which are part of innate immune system. If the nanoparticles are coated with antibiofouling molecules against RES, they will end up in several organs such as liver, spleen or lymph nodes. Even though this seems to be a disadvantage, RES mechanism can be successfully used for mapping liver, spleen and lymph nodes [13]. Alternatively, small linker molecules that covalently bind to the surface of SPIONs and bear more functionality for grafting “effector” molecules can be employed to prepare linker functionalized SPIONs as a nanocarrier platform. Xu et al. reported dopamine as a robust linker to SPIONs for conjugation of functional molecules [14,15]. De Palma et al. prepared functionalized SPIONs modified with a series of silane ligands bearing different functional groups for further development [16]. Jun et al. chose 2,3-dimercaptosuccinic acid (DMSA) as a molecular anchor for grafting cancer targeting herceptin to SPIONs for magnetic resonance imaging (MRI) cancer diagnosis [17]. Sousa et al. studied the aspartic acid and glutamic acid absorption on maghemite nanoparticles and opened up the possibility of amino acid derivative SPIONs as a novel nanocarrier [18].

Hyaluronan (hyaluronic acid, hyaluronate, HA) is a polysaccharide comprising repetitive disaccharide (β-d-glucuronic acid-(1→3)-β-*N*-acetyl-d-glucosamine) moieties [19]. HA, as one of the major components of extracellular matrix (ECM), and the action of HA degrading enzymes, termed hyaluronidases, are supposed to be involved in wound healing and tissue regeneration and as well as in inflammation and in cancer metastasis [20]. Experimental evidence showed that the interaction between HA and hyaladherins (HA receptors, i.e., CD44 and receptor for hyaluronan-mediated motility (RHAMM), CD168) promotes the progression of a number of cancers. CD44, a glycoprotein found overexpressed on the common types of tumor cell plasma membrane such as lung, colon and breast cancer, and is of particular interest in understanding the HA signaling pathway in tumor development [21]. Standard CD44, together with its isoform, is encoded by a single gene. Standard CD44 is composed of an extracellular binding domain for HA recognition, a stalk-like region located between the extracellular domain and transmembrane stretch, and a cytoplasmic tail which interacts with cytoskeleton molecules. For variant forms of CD44, variant exon products are inserted between the stalk-like region and the transmembrane region [22]. Evidence of elevated levels of HA in most solid tumors and their surrounding stroma is closely related to tumor migration and invasion [23]. Correspondingly, HA receptor CD44 were found to be overexpressed on most malignant cells, such as breast, ovarian, colon, lung, stomach cancer and leukemia [22]. Di Meo et al. have shown localized accumulation of HA conjugated n-propyl carborane in colorectal, ovarian and bladder cancer cells in vitro, which is encouraging for using HA as a targeting agent for boron neutron capture therapy [24]. Varghese et al. reported the enhanced and localized delivery of low *Mw* biphosphonate (BP) which was conjugated to the crosslinked HA hydrogel to CD44-positive colon carcinomas [25]. Thus, the overexpression of CD44 could be a good tool in drug delivery approaches using the receptor as an anchor to attach, through a ligand, prodrugs or nanomedicine-based delivery systems to increase the efficiency of anticancer drugs [26].

The present report aims to develop more stable conjugation protocol by covalently binding the targeting molecules (HA) with glutamic acid as a molecular linker and peripheral surface of SPIONs. Especially, to avoid the quick break down and clearance by liver, HA was introduced as both a protective substance to reduce the cytotoxicity of SPIONs and a targeting anchor to improve the translocation of SPIONs in cancer cells. The synthesis of HA-GA@SPIONs was included oxidization with H_2_O_2_ followed by activation of hydroxyl group and reacting glutamic acid as an intermediate molecule demonstrating transfection of lung cancer cells.

## 2. Results

### 2.1. Synthesis and Surface Modification of Superparamagnetic Iron Oxide Nanoparticles (SPIONs)

Glutamic acid (GA), a natural-rich amino acid that bears two carboxylic groups and one amino group, was employed as a linker molecule to graft biomacromolecules. It has been well established that the carboxylic species bind to metal oxide surface via chemical coordination instead of simple physical absorption. The chemisorption of carboxylic species onto iron oxide surface involves the replacement of hydroxyl groups and direct coordination of carboxylic groups with underlying Fe ions. There are four coordination modes for the complexion of carboxylic groups with metal oxides: ionic, monodentate, bidentate and bridging modes.

Here we have chosen hyaluronic acid (HA) as a model for conjugation of bio-molecules [27]. By using standard 1-ethyl-3-(3-dimethylamino) propyl carbodiimide, hydrochloride (EDC)/*N*-hydroxysuccinimide (NHS) protocol, a stable amide bond is formed between the amine functionalized GA-SPIONs and carboxylate of HA. HA has also received intensive investigation as a new drug carrier and tissue scaffold due to its biocompatibility, low immunogenicity and high viscosity [28,29,30]. However, only a few cases of HA-decorated nanoparticles have been reported, most of which are focused on the positive aspects of biocompatibility to improve the cytotoxicity of the core particles and are prepared by simple physical absorption [24,31]. It can certainly be claimed that conjugation of HA onto SPIONs via GA linkers is capable of producing more stable HA coated SPIONs in comparison with physical absorption. Moreover, HA plays an important role in promoting cell proliferation and migration due to its ability to absorb water and create a gel-like environment. HA also regulates cell signaling through receptor-specific interaction in both physiological and pathological conditions. Studies have demonstrated that HA receptors, such as CD44 and RHAMM, are overexpressed in various types of tumor metastasis [22]. In the present work, conjugation of HA onto SPIONs not only provide adequate colloidal stability and high biocompatibility, but also increases cellular uptake in cancerous cells for cell-type specific targeting in potential drug delivery or imaging applications.

Zeta-potential measurements of nanoparticles are a commonly used technique to confirm the presence of polysaccharide or other charged molecules on the surface of the particles [32]. After complete reaction of ammonia coprecipitation, magnetite nanoparticles are formed and dispersed in aqueous solution. At the iron oxide-water interface, the surface iron ions possess an unoccupied atomic orbital hence they react with water molecules or hydroxyl ions giving raise the surface Fe-OH functional groups. Since the surface hydroxyl groups have a double pair of electrons and dissociable hydrogen, B-SPIONs are amphiprotic. The absorption and desorption of protons on the B-SPIONs surface can be presented as followed:

(1) $$FeOH\;↔FeO^{-}+H^{+}$$

(2) $$FeOH+H^{+}↔FeOH_{2}^{+}$$

In acidic solution, the protonation of Fe–OH groups dominates where the majority of hydroxyl groups on the surface are in the form of FeOH_2_^+^ hence the B-SPIONs display a positive charge at low pH; while in a basic environment the dissociation of hydrogen dominates leaving negative FeO^−^ groups on the surface of B-SPIONs.

As discussed earlier, H_2_O_2_ treatment under hydrothermal conditions oxidizes the outer layer of magnetite into maghemite that has a higher density of surface hydroxyl groups. The increase of maximum positive/negative charge can be explained by the increased amount of surface hydroxyl groups.

**Figure 1:**
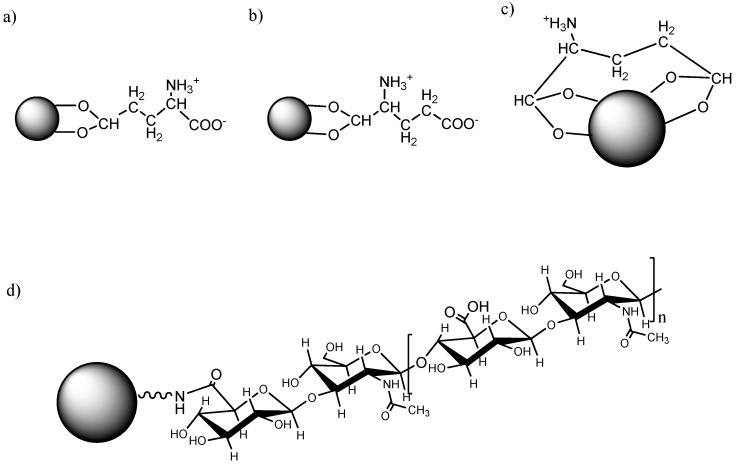
(**a**–**c**) Three possible configurations of glutamic acid superparamagnetic iron oxide nanoparticles (GA-SPIONs); and (**d**) schematic illustration of hyaluronic acid superparamagnetic iron oxide nanoparticles (HA-SPIONs).

The surface activated SPIONs were prepared by a step-wise approach for the attachment of biomacromolecules. Firstly, SPIONs with ~6 nm diameter were produced by coprecipitation, followed by hydroxyl activation and oxidization by H_2_O_2_. Glutamic acid (GA) was chosen as a small intermediate molecule for further conjugation of bio-effecters, due to its metal chelating properties and biocompatibility. After chemisorption of GA onto the surface of activated SPIONs, HA, a polysaccharide known to be associated with malignant cells, was conjugated to GA activated particles (GA-SPIONs) by a standard EDC-NHS protocol [33]. Since there are two carboxylic metal chelating functionalities in GA, it is possible that GA binds to the SPION’s surface via different configurations, as schematically presented in Figure 1a–c.

**Figure 2:**
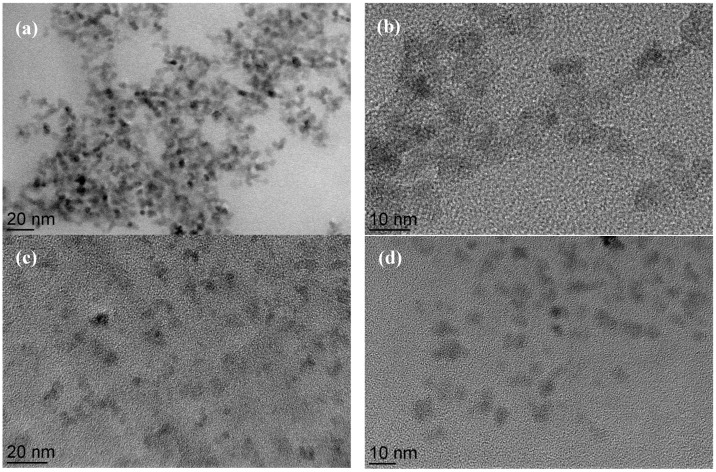
Transmission electron microscopy (TEM) images of: (**a**) bare SPIONs (B-SPIONs); (**b**) hydroxyl group activated SPION (OH-SPIONs); (**c**) GA-SPIONs; and (**d**) HA-SPIONs.

Figure 2 shows the transmission electron microscopy (TEM) to investigate morphology of the SPION core (B-SPION), dispersity and arrangement during the surface modification with GA and HA. Hydroxyl group activated SPION(OH-SPIONs) form a stable colloidal in water and have undefined morphology with a diameter of 10 nm, whereas bare SPIONs (B-SPIONs) exhibit irregular mixtures of spherical and cubic-shapes with a diameter of 7–8 nm together with high degree of agglomeration. The results imply that the formation of hydroxyl group by H_2_O_2_ treatment induce the negative surface charge on the surface of magnetic particles resulting in separation of particle as agglomeration is reduced. In addition, GA-SPIONs and HA-SPIONs are fully separated with clustering of 2–3 particles because the polymeric molecules could serve as a stabilizer to prevent aggregation.

### 2.2. Fourier Transform Infrared Analysis of Surface Modified SPIONs

**Figure 3:**
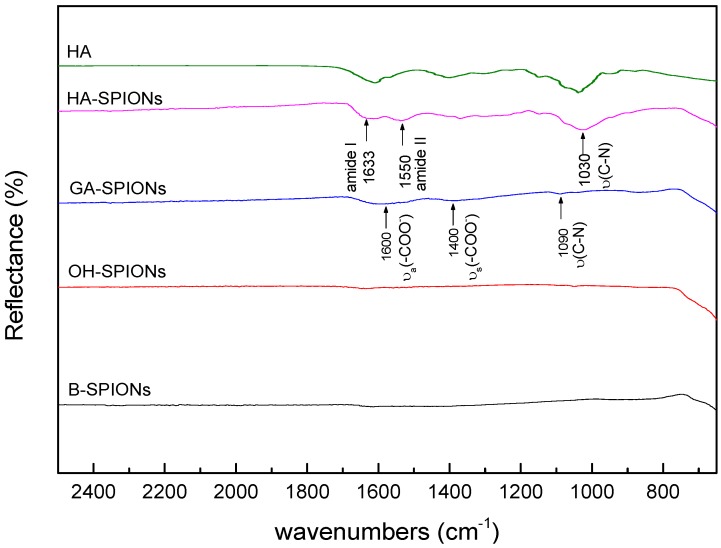
Fourier transform infrared spectra of B-SPIONs, OH-SPIONs, GA-SPIONs and HA-SPIONs in the wavenumber range of 2500 cm^−1^ to 650 cm^−1^.

Figure 3 shows the Fourier transform infrared (FTIR) spectra of B-SPIONs, OH-SPIONs, GA-SPIONs and HA-SPIONs. In the spectrum of B-SPIONs, there is a sharp peak observed at 546 cm^−1^ assigned to the Fe–O bond. In the region of 3500 cm^−1^ to 3000 cm^−1^, there is a very weak and broad band due to both physically absorbed water and surface hydroxyl groups, since the drying process does not remove all the water content. The spectrum of OH-SPIONs is similar to the spectrum of B-SPIONs; except there is a new peak appearing at 630 cm^−1^, which is a characteristic band for maghemite [34], indicating that part of the particles have transformed into a different phase. However, FTIR analysis is not capable of quantitative analysis of surface hydroxyl groups because the OH stretching of physically absorbed water interferes with the surface –OH stretching.

Because of the very small amount of GA that binds to the surface of iron oxide particles, it is difficult to distinguish small peaks in the spectrum. There are two relatively broad bands appearing at 1600 cm^−1^ and 1400 cm^−1^, assigned to the asymmetrical and symmetrical carboxylate vibrations, which are characteristic bands for GA in amino acid analysis [18]. It is possible to postulate the coordination mode of carboxylate to the metal oxide by determining the Δ, the difference between ν_a_(COO^−^) and ν_s_(COO^−^). In the case of GA-SPIONs, Δ = 200 cm^−1^, hence GA binds to the iron oxide surface through a bidentate mode. Two small peaks observed at 1090 cm^−1^ and 1040 cm^−1^ are assigned to C–N stretching modes [18].

There are two characteristic secondary amide bands appearing in the HA-SPIONs spectrum. The first one appears at 1633 cm^−1^, assigned to amide I bond, which is attributed to C=O stretching vibration of the amide coupled with the in-plane N–H bending and C–N bending modes. The amide II band appearing at 1550 cm^−1^, attributed to both N–H stretching and C–N stretching in the amide groups. The existence of carboxylate functionality in HA-SPIONs is also quite evident. It is known that part of carboxylate groups in HA form secondary amide bond with amino groups on the surface of GA-SPIONs, while the remaining carboxylic group exist in the form of sodium salts, since the C=O stretching band at 1700 cm^−1^ in –COOH group is absent [18]. There is a strong band at 1030 cm^−1^ due to C–N stretching in the primary amine. The presence of such bands indicates that the primary amine functionalities are available in HA-SPIONs, probably because not all amine groups in GA-SPIONs form a covalent amide bond with HA in solution. Hence there are both carboxylate and amine functionalities on the surface of HA-SPIONs, which enables easy access for grafting different imaging or diagnostics moieties onto HA-SPIONs.

**Figure 4:**
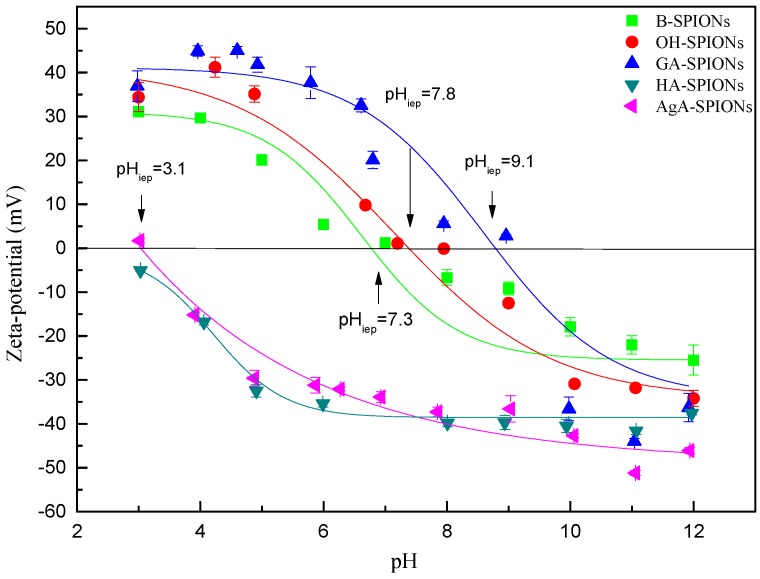
Zeta-potential of B-SPIONs, OH-SPIONs, GA-SPIONs, HA-SPIONs and agarose coated SPIONs (AgA-SPIONs).

To investigate the surface charges and influence of the coating on colloidal stability, the zeta-potential of particles were measured after each treatment. Figure 4 shows the zeta-potential curves of B-SPIONs, OH-SPIONs, GA-SPIONs, HA-SPIONs and agarose coated SPIONs (AgA-SPIONs) against pH. When the samples were kept in vacuum vial at 4 °C, the sample could be preserved for least six months without precipitation and color changes coming from phase transitions.

### 2.3. Surface Charge by Zeta Potential

The zeta-potential of B-SPIONs decrease when pH increases, with a maximum positive charge of +31.1 mV ± 1.8 mV at pH = 3 and a maximum negative charge of −25.5 mV ± 3.4 mV at pH = 12. The isoelectric point (IEP) of B-SPIONs was determined to be pH_iep_ = 7.3, which corresponds well with the reported value [35,36], OH-SPIONs show a very similar pH-dependency behaviors and pH_iep_ = 7.8 as B-SPIONs. The maximum positive charge was found to be +41.2 mV ± 2.3 mV at pH = 4, and the maximum negative charge of −34.2 mV ± 1.8 mV at pH = 12. At range of pH = 3~6, the zeta-potential of GA-SPIONs is stabilized around +40 mV. In the range of pH = 6~10, the zeta-potential is very sensitive to pH change. GA-SPIONs display a highly negative charge of −44.0 mV ± 0.7 mV, at pH = 11. The amine activation of GA-SPIONs allows attachment of carboxylic species by a standard EDC/NHS protocol. HA-SPIONs display a negative surface charge though the whole range of pH and the zeta-potential decreases with pH increase and show no IEP. HA (in the sodium salt form) possesses one carboxylate functional group that is negative. It has been reported that HA coated polycaprolactone (PCL) nanoparticles display a surface charge of −45 mV. In the case of HA-SPIONs, it is possible that all the amino groups are used in HA conjugation or HA masks the positive charge of amino groups in the core GA-SPIONs. Agarose (AgA) is also a negatively charged polysaccharide with carboxylate functionalities; the same conjugation protocol was applied to produce AgA-SPIONs. The IEP of AgA-SPIONs was determined to be pH_iep_ = 3.1, however even at pH = 3, AgA-SPIONs demonstrated good colloidal stability, indicating that AgA stabilizes the nanoparticles by steric repulsion. GA has two metal chelating carboxylic groups and one amine group. Therefore, after chemisorption of GA onto the surface of OH-SPIONs, one carboxylic group coordinate with the surface Fe atom on SPIONs, leaving both remaining carboxylic groups and an amine group on the surface. At acidic pH, amine groups are easily protonated to display positive charges, and at basic pH, the ionization of carboxylic groups to form carboxylate dominates in the system, hence, a negative charge of GA-SPIONs is observed. The right-shift of IEP to pH_iep_ = 9.1 and high value of positive/negative surface charges are good indicators of adequate GA chelating.

### 2.4. Intercellular Uptake

**Figure 5:**
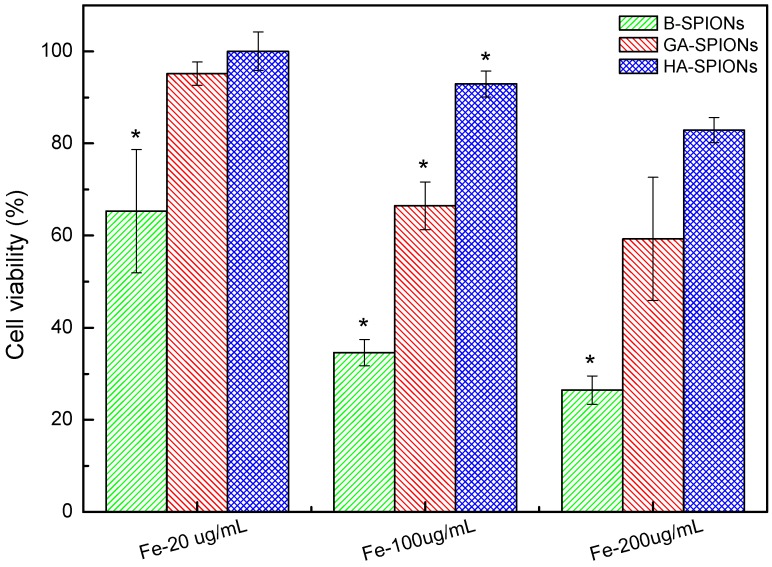
Histogram of cell viability after 24 h incubation with B-SPIONs (green), GA-SPIONs (red) and HA-SPIONs (blue) at 37 °C, 5% CO_2_. (* means the mean of the group is significantly different from the other two at the same Fe concentration at *p* = 0.05 level) (*n* ≥ 5).

Figure 5 shows the cell viability after 24 h incubation of HA-SPIONs and GA-SPIONs at 37 °C, 5% CO_2_. At low concentrations (20 µg/mL), both HA-SPIONs and GA-SPIONs do not affect cell viability. However, at higher iron concentration (100 µg/mL and 200 µg/mL), the viability of HA-SPIONs incubated cells was reduced to 87% and 83%, respectively, which is acceptable. For GA-SPIONs, the cell viability significantly reduced to 66% and 63%, respectively. Such results suggested that GA as a linker molecule for grafting HA onto SPIONs is sufficient, but GA-SPIONs are not suitable for direct use in nanomedicinal applications. From zeta-potential analysis, the surface charge of GA-SPIONs at physiological pH was determined to be around +12 mV. This means GA-SPIONs may form aggregates at pH = 7.4. Because electrostatic repulsion is not enough to disperse the particles and GA molecules are too small to have a steric repulsion. Moreover, MTT results showed that the cytotoxicity of GA-SPIONs cannot be neglected. Once HA is grafted onto the SPIONs, HA not only acts as a coating to separate individual particle sterically, but also improves the biocompatibility of SPIONs.

**Figure 6:**
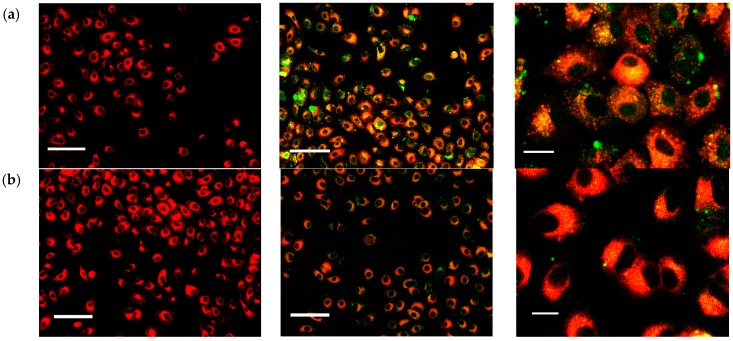

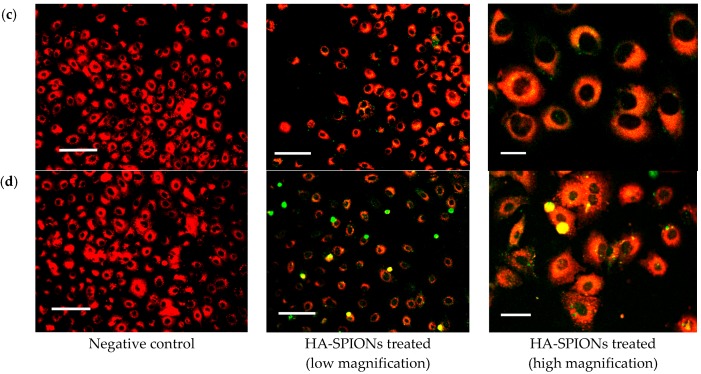
Confocal microscopy images of A459 cells only as control (**left** column); A459 cells incubated with 20 µg/mL fluorescein isothiocyanate (FITC)-HA-SPIONs (green colored) (**middle** column) and enlarged imaged of A459 cells with FITC-HA-SPIONs (**right** column) for: (**a**) 6 h; (**b**) 8 h; (**c**) 12 h; and (**d**) 24 h. Cells were stained with red membrane dye PKH26 prior to FTIC-HA-SPIONs incubation at 37 °C, 5% CO_2_ (the scale bars of the left and middle column are 100 mm, and the scale bars of the right column are 20 mm).

**Figure 7:**
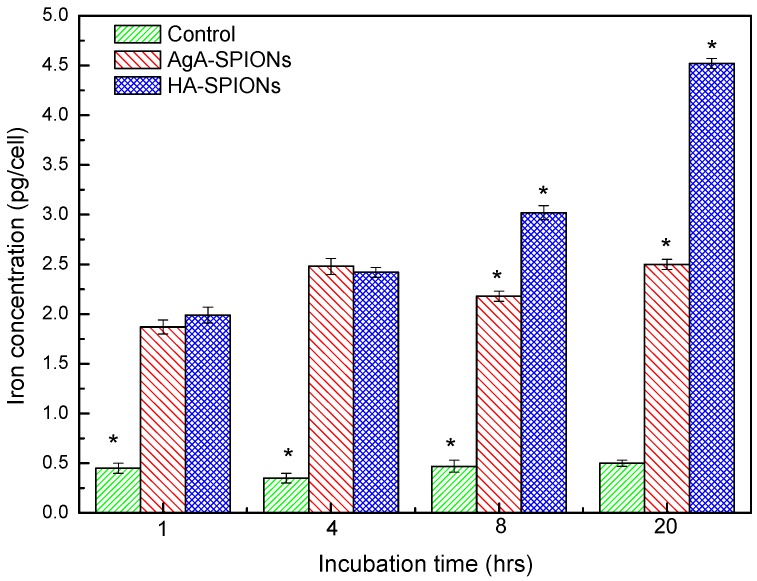
Histogram of cellular iron uptake of A459 cells without any treatment (green) and those incubated with AgA-SPIONs (red) and HA-SPIONs (blue), cells with no particles treatment as control (green), at 1 h, 4 h, 8 h and 20 h incubation, at 37 °C, 5% CO_2_. (* means the mean of the group is significantly different from the other two groups at *p* = 0.05 level) (*n* ≥ 5).

To investigate the intracellular behaviors of HA-SPIONs, the cellular uptake in living cells was examined by confocal microscopy. Figure 6 shows representative fluorescent overlay images of A459 cells after incubation with fluorescein isothiocyanate (FITC) conjugated HA-SPIONs for different time intervals. PKH 26 was used as a membrane specific dye to stain cells, for observation of cell morphologies. HA-SPIONs treated cells for up to 20 h shows no significant morphological changes in comparison with A459 negative controls, indicative of very low or no adverse effect of such particles with cells. This is consistent with the results of MTT assays. HA-SPIONs firstly formed bright small clusters around the cells very quickly after 6 h incubation with A459 cells, suggesting that HA-SPIONs are attached to the membrane-bound HA receptors, e.g., CD44, which is found to be over-expressed on A459 cell surface, in a multivalent configuration. At 8 h interval, it can be clearly seen that the green fluorescence is evenly distributed inside the cells. HA-SPIONs bind to CD44 receptors on the cell surface, followed by endocytosis into the cytoplasm. In Figure 7, histograms of SPION cellular internalization were shown as a function of incubation time. Control samples were A459 cells without any nanoparticle incubation, the iron content was determined to be ~0.40 pg/cell, regardless of incubation time. AgA-SPIONs treated A459 cells showed increased iron content to 2.48 ± 0.08 pg/cell at 4 h incubation, which indicated that non-specific SPIONs endocytosis occurred. A completely different uptake pattern is shown with HA-SPIONs treated cells: at 1 h and 4 h incubation, the cellular iron content (1.87 ± 0.07 pg/cell and 2.48 ± 0.08 pg/cell, respectively) are similar to AgA-SPIONs group (1.99 ± 0.08 pg/cell and 2.42 ± 0.05 pg/cell respectively), which can be explained by the fact that HA-SPIONs start to bind to the CD44 receptors and are accumulated at the cell surface. During the incubation, the cellular iron content of HA-SPIONs group increases, suggesting that more particles bind to surface receptors with HA-CD44 interaction and receptor-mediated endocytosis proceeds. Cellular iron content reached 4.52 ± 0.05 pg/cell after 20 h incubation, while the iron content of AgA-SPIONs group remained 2.50 ± 0.05 pg/cell. These results are consistent with confocal microscopy analysis results described earlier.

It is well known that HA is very sensitive to environmental pH, and the degradation occurs very intensively when the pH is less than pH = 4 or more than pH = 10 [30]. In acidic endosome/lysosomes, it is possible that the low pH degrades the HA coating into oligomers, thus breaking down the small aggregates of HA-SPIONs to improve the monodispersity of the particles. This can lead to leakage of small fragments of fluorescently labeled HA, which is why the evenly distributed green fluorescence is observed. In addition, the acidic pH inside the endosomes also assists the dissociation of HA and its receptors, therefore changing the configuration and dispersion of HA-SPIONs to display non-cluster like fluorescence. In conclusion, HA-SPIONs are internalized into A459 cells via receptor-specific endocytosis. Agarose (AgA), another type of negatively charged polysaccharide, which has a similar molecular structure to HA, but different binding properties, can act as a control coating substance to identify the cancer-specific targeting property of HA-SPIONs.

## 3. Materials and Methods

### 3.1. Materials and Instrumentation

Iron (II) chloride tetrahydrate (FeCl_2_∙4H_2_O, 99%), iron (III) chloride hexahydrate (FeCl_3_∙6H_2_O, 99%), aqueous ammonia (30%, *v*/*v*), glutamic acid (GA, 99.9%), hyaluronan sodium salts (HA, Mw = 0.6–1.1 MDa, polydispersity = 1.02, 99%), hydrogen peroxide (H_2_O_2_, 30%, *v*/*v*) were purchased from Sigma-Aldrich (Seoul, Korea). Double distilled H_2_O (ddH_2_O) was used throughout chemical synthesis and sample preparation for characterization. Sterilized ddH_2_O was used for cell-related experiments.

Dulbecco’s Modified Eagles Medium (DMEM), RPMI medium, fetal bovine serum (FBS), 200 mM L-glutamine, penicillin/streptomycin and amphotericin B were purchased from Biosera (NUAILLE, France). 3-(4,5-dimethylthiazol-2-yl)-2,5-diphenyl tetrazolium bromide (MTT) assay kit and luciferase assay kit were purchased from Promega (Madison, WI, USA). The size and morphology of nanoparticles was examined on two transmission electron microscopes model JEOL 2100F (200 kV) and 1230 (120 kV, Tokyo, Japan), e.g., the physical sizes of iron oxide cores were measured on JEOL 2100F (200 kV); and both core and shell structures were observed under JEOL 1230 (120 kV). TEM samples were prepared by placing a few drops of nanoparticle suspension onto a carbon-coated grid and air drying under ambient conditions. For physical size measurement, the diameter of >100 nanoparticles were measured on digital TEM images using image analysis software ImageJ. The hydrodynamic size of nanoparticles was determined by photon correlation spectroscopy (PCS), using a Zeta-Sizer HA300 (Malvern, Worcestershire, UK). Samples were dispersed in ddH_2_O with iron concentration of ~10 μg/mL. For zeta-potential measurement, all samples were diluted with ddH_2_O into ~10 μg/mL iron concentration, and the pH was adjusted with 0.1 N NaOH or 0.1 N HCl to the range of pH = 3 to pH = 12, respectively. Fourier transform infrared (FTIR) spectra were recorded at 20 °C using an Alpha FTIR Spectrometer equipped with Platinum ATR (single reflection diamond attenuated total reflectance) from Bruker Optics (Rosenheim, Germany). The samples were dried in oven at 50 °C overnight and grinded into fine powder with a motor and pestle prior to measurement. Spectra were measured with a resolution of 1 cm^−1^ and the wavenumber range was 500–4000 cm^−1^.

### 3.2. Peroxide Activation, Glutamic Acid (GA) Modification and Hyaluronan Sodium Salts (HA) Conjugation to Bare SPIONs (B-SPIONs)

Bare SPIONs (B-SPIONs) were synthesized by alkaline coprecipitation**.** ddH_2_O was deoxygenated with N_2_ gas for 20 min prior to all experiments. Then, 33.79 g FeCl_3_·6H_2_O and 12.43 g FeCl_2_·4H_2_O were dissolved in 250 mL ddH_2_O to make iron stock solution ([Fe^3+^] = 0.5 M, [Fe^2+^] = 0.25 M). Next, 1.6 mL iron stock solution (total Fe content 1.2 mmol) was made up to 38 mL with ddH_2_O at 4 °C. Two milliliters of ice cooled NH_4_OH was poured into the iron solution under vigorous magnetic stirring, during which black precipitates appear immediately. The reaction mixture was heated to 70 °C for 30 min to complete the reaction. The black precipitate was collected with a rare earth magnet and washed three times with ddH_2_O to remove the unreacted ions and salt. Finally, as-prepared SPIONs was re-dispersed in ddH_2_O via sonication and stored at 4 °C for further experiments.

One hundred microliters of H_2_O_2_ was added to 10 mL of B-SPIONs (containing 5 mg/mL Fe). The mixture was heated to 80 °C and kept there for 20 min until there was no gas evolution. The black colored B-SPIONs gradually turned into a reddish brown suspension. The hydroxyl activated SPIONs (referred as OH-SPIONs) were sonicated for 10 min in an ice bath to re-disperse in ddH_2_O and stored at +4 °C.

One hundred milligrams of glutamic acid (GA) was added to 10 mL of OH-SPIONs (containing 5 mg/mL Fe) and stirred at room temperature for 3 h, during which GA chemi-absorbed onto the peroxide activated surface. The reddish brown suspension was subjected to dialysis against 5 L ddH_2_O for 2 days to remove unbound GA.

Ninety-six milligrams of 1-ethyl-3-(3-dimethylaminopropyl)carbodiimide (EDC) and 15 mg *N*-hydroxysuccinimide (NHS) were added to 3 mL HA solution (5 mg/mL), and the mixture was added to 5 mL GA-SPIONs (containing 3 mg/mL Fe). The reaction mixture was shaken at room temperature for 4 h. The HA conjugated particles were dialyzed against 5 L ddH_2_O for 2 days to remove free HA. HA-SPIONs suspensions were stored at +4 °C.

Agarose conjugated SPIONs (AgA-SPIONs) were prepared under the same conditions. Three milliliters of agarose solution (5 mg/mL, heat the agarose solution at 60 °C to dissolve) was reacted with 96 mg EDC and 15 mg NHS, and the mixture was added to 5 mL GA-SPIONs (containing 3 mg/mL Fe). Finally, AgA-SPIONs were dialyzed against 5 L ddH_2_O for 2 days to remove free agarose. AgA-SPIONs were stored at +4 °C.

### 3.3. Cytotoxicity by 3-(4,5-Dimethylthiazol-2-yl)-2,5-diphenyl Tetrazolium Bromide (MTT) Assays

A459 Cells were cultured in DMEM supplemented with 10% fetal bovine serum (FBS), 2 mM l-glutamine and 1% penicillin/streptomycin at 37 °C in a humidified atmosphere at 5% CO_2_ in 25 cm^2^ cell culture flasks. Medium was changed every two or three days until 90% confluence was achieved.

The cytotoxic effect of HA-SPIONs, and AgA-SPIONs at various concentrations for up to 5 day incubation time were assessed by 3-(4,5-dimethylthiazol-2-yl)-2,5-diphenyl tetrazolium bromide (MTT) assay. MTT assay was a cell proliferation assay based on the ability of a mitochondrial dehydrogenase enzyme in viable cells to cleave the tetrazolium rings of the pale yellow MTT and form a dark blue formazan crystal. The number of surviving cells was directly proportional to the level of the formazan product created, which can then be quantified by reading absorbance at 570 nm. Cells were incubated with HA-SPIONs, and AgA-SPIONs containing media at desired iron concentrations for various time intervals. Ten microliters of MTT solution was added to the medium and the mixtures were incubated for 4 h at 37 °C, followed by addition of 100 μL dimethyl sulfoxide (DMSO) to dissolve the purple crystals with gentle pipetting. Absorbance was measured at 570 nm. Control cells were cultured with complete medium only. Each experiment was carried out in triplet and repeated twice. The results were analyzed and plotted in Origin 7.0; one-way analysis of variance (ANOVA) was performed to compare the means of each sample groups, in which Tukey test was used as means comparison test, and Levenell test was used as equal variance test.

### 3.4. Cytotoxicity by Live/Dead Two-Colored Cell Viability Assay

The cytotoxicity of HA-SPIONs, and AgA-SPIONs was verified by live/dead viability staining kit (Molecular Probes) and visualized by confocal microscopy. The live/dead viability kit provided a two-color fluorescence cell viability test based on intracellular esterase activity and plasma membrane integrity. Intracellular esterase activity, a unique feature of live cells, was determined by the conversion of non-fluorescent calcein acetoxymethyl (AM) into green fluorescence calcein that was well retained inside the live cells to produce a strong green fluorescence. Weakly red fluorescent ethidium homodimer-1 (EthD-1) was actively excluded by the intact plasma membrane of live cells, while the molecules can enter the cellular membrane of dead cells and bind to nucleic acid to produce a 40 times stronger red fluorescence inside the dead cells. Cells were seeded in a 24-well plate at 2 × 10^4^ cells/well 20 h prior treatments with HA-SPIONs, and AgA-SPIONs for cell attachment. One hundred microliters of HA-SPIONs and AgA-SPIONs for various time intervals up to 5 days. After appropriate treatment of cells, 100 µL 2 µM calcein AM and 4 µM EthD-1 solution (in PBS) was added to each well to completely cover the surfaces, and incubated for 30 min in dark at room temperature. The stained cells were immediately examined under a laser scanning confocal microscope (Olympus). Three cell images were collected from each sample, and no post-acquisition enhancing processing was performed. The experiment was repeated twice at least.

### 3.5. Cellular Internalization Study

A459 Cell membrane was stained by PKH26 red fluorescence cell linker kit (Sigma, Seoul, Korea) according to the standard protocol supplied by the manufacturer. Briefly, A459 cells were trypsinized, collected and counted on a hemocytometer. Next, 2 × 10^7^ cells were washed once with medium without serum in a polypropylene tube and collected by centrifuge at 400 rpm for 5 min. The cell pellet was re-suspended in 1 mL diluent C and quickly mixed with 1 mL freshly prepared 4 × 10^−6^ mole PKH26 dye in diluent C in a polypropylene tube and incubated for 5 min at 25 °C. Two milliliters of FBS was added to the mixture to stop the reaction, followed by addition of 4 mL complete medium. Cells were centrifuged at 400 rpm for 10 min at 25 °C and the loose pellet was washed with complete medium a further three times. Finally, 10 mL complete medium was added to the washed cell pellet and cells were counted again on a hemocytometer. Cells were plated at 5000 cells/well in a 24-well flat-bottom plate in 2 mL complete cell culture medium in each well for 24 h at 37 °C, 5% CO_2_.

FITC-tagged HA-SPIONs, and AgA-SPIONs were added to A459 cell cultures and incubated in the dark at 37 °C, 5% CO_2_ atmosphere for different time intervals. Excess and unbound particles were removed by washing in PBS three times. Cells were fixed by 4% glutaraldehyde in PBS at 25 °C for 20 min. Three cell images were collected from each sample, under the 10× objective lenses, and no post-acquisition enhancing processing was performed. The experiment was repeated twice. The number of green dots and red viable cells in each microscopy image was counted and analyzed. The cellular uptake of FITC-tagged particles can be expressed as:

Cellular uptake (%) = green dots/red dots × 100%(3)

### 3.6. Quantitative Analysis of Particle Cellular Internalization

A459 Cells were seeded in a 6-well plate at 2 × 10^5^ cells/well 20 h prior treatments with HA-SPIONs and AgA-SPIONs. After incubation with HA-SPIONs and AgA-SPIONs at varied time intervals, A459 cells were washed by PBS for 3 times and trypsinized by trypsin/EDTA at 37 °C for 5 min. The particle-treated cells were collected by centrifuge at 10,000 rpm for 5 min. The cell number of each sample (collected from ≥4 wells) was estimated by cell counting with a hemocytometer under optical microscope. The cell pellet was dried in a heat block at 70 °C for 1 h and dissolved in 200 µL concentrated HCl for 4 h to ensure the complete dissolution of iron oxide nanoparticles.

### 3.7. Determination of Fe Content

The determination of Fe content was modified based on a published protocol [37]. Standard iron solution was prepared by diluting increasing amount of iron stock solution (total [Fe] = 0.75 M) (20, 40, 60, 80 and 100 µL) with ddH_2_O and adjust total volume to 150 µL A mixture of 20 µL concentrated HCl, 20 µL 10% NH_2_OH, 200 µL ammonium acetate buffer, 80 µL 0.1% 1,10-phenanthroline solution and 1030 µL ddH_2_O was added to each standard iron solution and incubate at R.T. for 20 min. (ddH_2_O was used as blank sample.) The absorbance of a series standard iron solution at 510 nm was measured by a UV-Vis spectrometer TG80, and plotted against blank as a function of Fe concentration of known standard iron solution. The iron concentration standard curve was obtained by using linear regression as an approximate function.

Twenty-microliter aliquots of SPION suspension were completely dissolved in 200 µL concentrated HCl, until the color of suspension was turned from brownish to very pale yellowish, adjust the volume of the mixture to 2 mL with ddH_2_O. Then, 150 µL of the resultant solution was added to a mixture of 20 µL concentrated HCl, 20 µL 10% NH_2_OH solution, 200 µL ammonium acetate buffer, 80 µL 1% 1,10-phenanthroline solution and 1030 µL ddH_2_O and incubated at room temperature for 20 min. The absorbance of sample at 510 nm was measured by UV-Vis spectrometer TG80, and fitted into the iron concentration standard curve shown, to calculate the iron concentration of the SPION suspension. The absorbance of standard iron solution at 510 nm was measured and the iron concentration standard curve was plotted every time prior to measurement of absorbance of sample SPION suspension at 510 nm.

## 4. Conclusions

In this work, hyaluronan (HA), a naturally occurring polysaccharide, was selected as both protective coating substances and targeting ligand for SPIONs. Instead of physical absorption, glutamic acid was used as a molecular linker to covalently conjugate HA onto SPION surfaces. FTIR and zeta-potential studies confirmed the chemical bonding between amino acid linker and polysaccharides. MTT cytotoxicity assay showed that HA-SPIONs treated cells remained 82.9% ± 2.7% alive at high particle dosage (200 µg/mL iron concentration), whereas GA-SPIONs and B-SPIONs treated cells had only 59.3% ± 13.4% and 26.5% ± 3.1% survival rate at the same conditions. Confocal microscopy analysis showed that increased cellular internalization of HA-SPIONs compared to non-interacting AgA-SPIONs. Fe content analysis showed the cellular iron uptake in A459 cells reached 4.52 ± 0.05 pg/cell after 20 h incubation with HA-SPIONs, 40% higher than AgA-SPIONs treated cells.
